# Rationalization of Orthopaedic Surgical Instrument Trays: Three Years’ Experience of a Practical Approach to Cut Down Unnecessary Costs

**DOI:** 10.7759/cureus.19866

**Published:** 2021-11-24

**Authors:** Shady Hermena, Francesca Solari, Robert Whitham, Cara Hatcher, Oliver Donaldson

**Affiliations:** 1 Trauma and Orthopaedics, Yeovil District Hospital NHS Foundation Trust, Yeovil, GBR; 2 Trauma and Orthopaedics, Great Western Hospital NHS Foundation Trust, Swindon, GBR

**Keywords:** reducing the cost in orthopedic surgery, orthopedic surgical trays rationalization, trauma and orthopaedic, cost-effective practice, surgical instruments trays rationalization

## Abstract

Background

This study aimed to rationalize the surgical instrument trays (SITs) used in some trauma and orthopedic (T&O) procedures to reduce unnecessary costs.

Methods

SITs for several T&O procedures at our trust were assessed to judge the utility of each instrument. SITs for hip, knee, and shoulder arthroscopy, dynamic hip screw (DHS), rotator cuff repair, shoulder stabilization, total shoulder arthroplasty (TSR), and proximal humerus fracture fixation were reviewed. Infrequently used and irrelevant instruments were removed to minimize the number of used trays for each procedure. A qualitative survey was conducted following SIT rationalization to assess the practicality and suitability of these changes.

Results

The number of SITs was rationalized from four to two for DHS, three to one for hip, knee, and shoulder arthroscopy, five to two for rotator cuff repair and shoulder stabilization, three to one for TSR, and proximal humerus fracture fixation. Based on the local database figures for these procedures, the estimated number of used trays reduced from 2,785 to 1.015 (36.4%) trays per year. Based on the sterilization cost of £35 per tray, annual savings amounted to about £61,950. Qualitative analysis of theatre staff feedback showed increased time efficiency and a positive feeling of practicality.

Conclusion

The critical appraisal of the departmental operating practice is an effective tool to achieve cost-efficient practice. The rationalization of SITs for orthopedic procedures can result in significant savings by reducing sterilization costs alone.

## Introduction

Surgical procedures necessitate equipment sterilization for safe use and infection prevention [1]. Surgical instruments for each procedure are kept in surgical instrument trays (SITs). After each use, these SITs must be cleaned, disinfected, and sterilized by the Central Sterilisation Service Department (CSSD) to be ready for the following procedure [2,3]. Costs involved in orthopedic surgery range from staffing, theatre space, implants, surgical equipment, sterilization costs, and radiography equipment [4]. The hospital's operating theatres are the most expensive parts of a hospital to run [5].

The National Health Service (NHS) in the UK has been under increasing pressure in recent years to maintain high standards of care for its patients: from rising Accident and Emergency (A&E) attendances, admissions, referrals to secondary care, falling numbers of beds and a reduction in the funding as a percentage of the gross domestic product [6-8]. Austerity measures brought about as a result of the 2008 financial crisis had reduced the annual increase in NHS funding to 1.4% per year from 2009 to 2019, compared to the average annual increase of 3.7% from when the NHS was established, despite an increase in inpatient admissions of 3.6% per year [8,9]. In 2015/16, it was reported that NHS Trusts were at a £2.6bn deficit [8].

Trauma and orthopedic (T&O) procedures account for 25% of the UK's surgical workload [10]. The specialty has seen an increase in referrals from General Practice at a rate of 7%-8% per year [10]. It was also disproportionately affected during the coronavirus 2019 (COVID-19) pandemic, with 82% of its operations canceled globally during the first wave [11]. The COVID-19 pandemic resulted in approximately 86,000 cancellations for elective orthopedic operations and has contributed to 140,000 patients waiting over a year for elective operation in the UK [12,13]. At an average cost of £4000 per operation, it would cost £2bn to clear the elective backlog of operations caused by the pandemic [11].

With increasing demands on surgical services, there is a need for expeditious sterilization and preparation of SITs to help maximize theatre time. In the UK, there are approximately 1.2 million T&O procedures per year [14]. Innovative methods to improve efficiency and cost savings could be crucial in helping to relieve the burden. A recent systematic review of the topic has shown that there are cost savings to be made with SIT rationalization, as well as saving time in sterilizing each tray and allowing more trays to be sterilized at one time through a reduction in tray weight and number per operation [3]. This study aimed to introduce a rationalization program of T&O SITs at our unit to reduce costs and improve efficiency.

## Materials and methods

In December 2017, each orthopedic surgical tray for several standard T&O procedures was reviewed and evaluated by a consultant orthopedic surgeon and senior scrub nurse, and a checklist was developed for each at our institution (Yeovil District Hospital). This process included the SITs for basic hip, knee, and arthroscopic shoulder procedures, dynamic hip screw (DHS) fixation, shoulder rotator cuff repair, shoulder stabilization, total shoulder arthroplasty (TSR), and proximal humerus fracture open reduction, internal fixation (ORIF). Each SIT for these procedures was assessed to identify the current instruments' reusability and missing instruments of high demand that were required to be added to trays. Surgical instruments that were found to be irrelevant or rarely used during these surgical procedures were removed to minimize the number of SITs required per case.

The number of surgical trays before and after the rationalization process for the included procedures were identified from the CSSD records in our trust. A search of our local operating theatre database was used to calculate the yearly figures for each procedure. A figure of 35 pound sterling (£) was quoted as the cost of one surgical tray sterilization by our CSSD, and this was used to calculate cost savings resulting from the rationalization process.

Three years following SIT rationalization, a follow-up survey using Google Docs™ was conducted to obtain feedback from orthopedic surgeons and scrub nurses to evaluate the long-lasting practicality and efficiency of the rationalization process (Table 1).

**Table 1:** Online feedback survey distributed to local orthopedic surgeons and scrub nurses DHS = dynamic hip screw; ORIF = open reduction, internal fixation

	Question	Response
Q1	Reducing the number of trays for DHS is practical and reduces unnecessary instruments.	Likert scale from 1 to 5: "1", strongly disagree; "5", strongly agree
Q2	Do you require any further instruments during the DHS surgery (apart from the implant)?	Never, rare, occasionally, frequently, very often
Q3	Reducing the number of trays for arthroscopy is practical and reduces unnecessary instruments.	Scale from 1 to 5: "1", strongly disagree; "5", strongly agree
Q4	With the current arthroscopy trays, do you require any further instruments during the surgery (apart from the implant)?	Never, rare, occasionally, frequently, very often
Q5	Reducing the number of trays for shoulder arthroplasty is practical and reduces unnecessary instruments.	Scale from 1 to 5: "1", strongly disagree; "5", strongly agree
Q6	With the current shoulder arthroplasty trays, do you require any further instruments during the surgery (apart from the implant)?	Never, rare, occasionally, frequently, very often
Q7	Reducing the number of trays for proximal humerus ORIF is practical and reduces unnecessary instruments.	Scale from 1 to 5: "1", strongly disagree; "5", strongly agree
Q8	With the current proximal humerus ORIF trays, do you require any further instruments during the surgery (apart from the implant)?	Never, rare, occasionally, frequently, very often
Q9	Do you recommend including hemi-hip arthroplasty, total hip replacement, and total knee replacement in the rationalization process?	Scale from 1 to 5: "1", strongly disagree; "5", strongly agree
Q10	Any further required developments or comments?	

## Results

SIT number post-rationalization

After surgical trays were examined and instrument utility assessed, the number of SITs for DHS was rationalized from four to two trays. Hip, knee, shoulder, and ankle arthroscopy trays were both reduced from three to one. Rotator cuff repair and shoulder stabilization trays were rationalized from five to two trays. Open shoulder procedures trays, including shoulder arthroplasty and proximal humerus fracture fixation, were rationalized from three to one for each procedure (Table 2). The total number of SITs rationalized was reduced from 66 trays to 25 (a 62.12% reduction) trays for the included procedures.

**Table 2:** SIT number for each procedure pre- and post-rationalization SIT = surgical instrument tray

Procedure	Previous number of SITs	Number of SITs after rationalization
Dynamic hip screw	4	2
Hip, knee, shoulder, and arthroscopy	3	1
Rotator cuff repair/shoulder stabilization	5	2
Open shoulder procedures (proximal humerus fixation and shoulder replacement)	3	1

Cost implications resulting from SIT rationalization

From December 2017 to December 2020, 2505 procedures involving SITs from the rationalization process were performed. The cost for SIT sterilization for these procedures without rationalization would have been £292,425. After rationalizing SIT sterilization, the actual cost was £106,575, amounting to a 63.55% reduction. The total amount of saving over three years was £185,850. Table 3 summarises the potential cost before SIT rationalization and the resultant savings.

**Table 3:** Number of procedures performed over three years, the potential costs and actual savings after the rationalization of SITs SIT = surgical instrument tray; DHS = dynamic hip screw; TSR = total shoulder arthroplasty; ORIF = open reduction, internal fixation

Procedure		Number of procedures performed in 3 years	Potential sterilization cost without rationalization	Actual cost due to rationalization	Savings
DHS		240	£33,600	£16,800	£16,800
Arthroscopy	Knee	825	£86,625	£28,875	£57,750
	Shoulder	540	£56,700	£18,900	£37,800
	Ankle	120	£12,600	£4,200	£8,400
	Shoulder	180	£18,900	£6,300	£12,600
Rotator cuff repair/shoulder stabilization	Rotator cuff repair	240	£42,000	£16,800	£25,200
	Stabilization	60	£10,500	£4,200	£6,300
Open shoulder	TSR	120	£12,600	£4,200	£8,400
	Proximal humeral ORIF	180	£18,900	£6,300	£12,600
Total		2,505	£292,425	£106,575	£185,850

Survey feedback analysis

**Figure 1:**
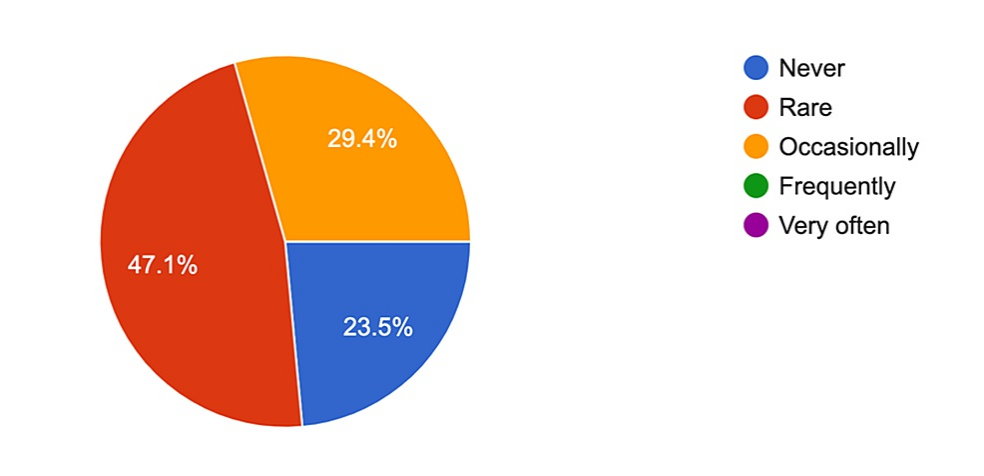
Responses for survey question 2: “With the current DHS trays, do you require any further instruments during the surgery (apart from the implant)?” DHS = dynamic hip screw

The SIT rationalization feedback survey was sent to 25 orthopedic surgeons and scrub nurses in our institution. The survey was completed by six consultants, six orthopedic registrars, and six scrub nurses. The response rate for the survey was 76% (n = 18). The mean response for DHS SIT instrument reduction and practicality was 4.62 (5 = strongly agree). The number (n) of survey respondents who felt they never required other instruments during the DHS surgery with the current trays after rationalization was four (23.5%); 47.1% (n = 8) participants felt they rarely require more instruments and 29.4% (n = 5) responded that they occasionally need more instruments with the rationalized DHS trays (Figure 1).

**Figure 2:**
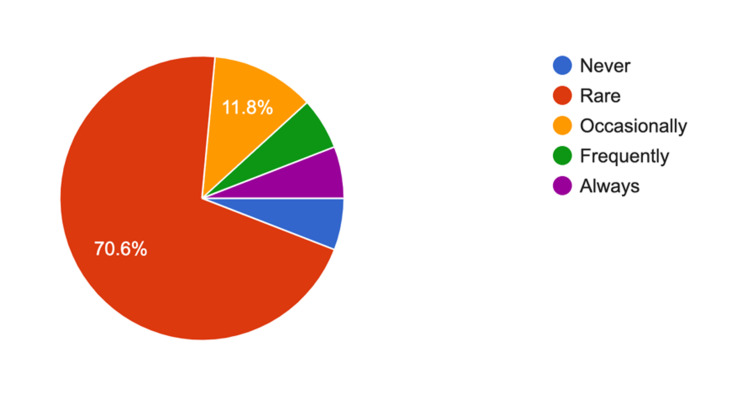
Responses for survey question 4: “With the current arthroscopy trays, do you require any further instruments during the surgery (apart from the implant)?”

**Figure 3:**
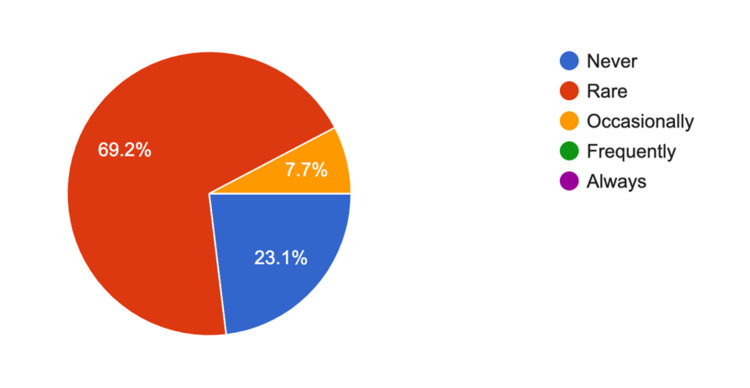
Responses for survey question 6: “With the current shoulder arthroplasty trays, do you require any further instruments during the surgery (apart from the implant)?”

**Figure 4:**
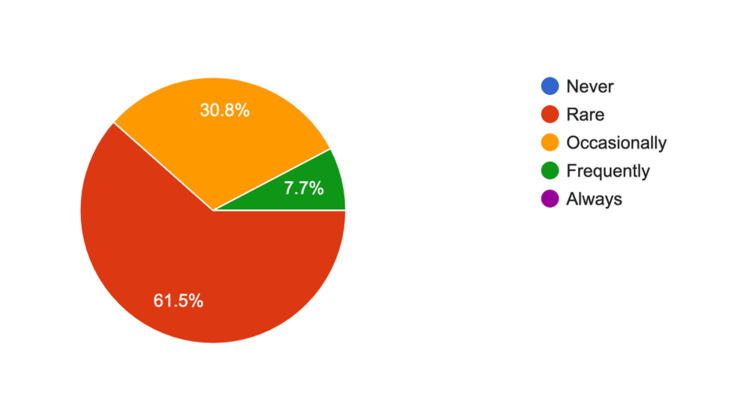
Responses for survey question 8: “With the current proximal humerus ORIF (open reduction, internal fixation) trays, do you require any further instruments during the surgery (apart from the implant)?”

The survey results for arthroscopy trays showed a 4.56 average response rate (5 = strongly agree) showing reducing the number of trays was practical and reduced unnecessary instruments; 70.6% (n = 12) of survey participants "rarely" needed further instruments of equipment (Figure 2). Similarly, for shoulder arthroplasty, the average response rate was 4.76 out of 5 for whether rationalizing the trays was practical and reduced unnecessary equipment, and 69.2% of respondents (n = nine) rarely needed additional equipment (Figure 3). For proximal humerus ORIF trays, the Likert scale response was 4.91 about the practicality of rationalization, with 61.5.% (n = 8) rarely needing any additional equipment, 30.8.% (n = 4) occasionally and 7.7% (n = 1) frequently needing further equipment (Figure 4). Furthermore, 16 participants (88.8%) either strongly agreed or agreed to include hemi-hip arthroplasty, total hip replacement, and total knee replacement in the rationalization process.

## Discussion

With the NHS's financial challenges, there is a continuous need for a cost-effective practice without compromising patients' safety. Significantly, as T&O procedures account for 25% of surgical procedures in the UK, any improvements in efficiency and cost savings in our specialty will help overall as we occupy a high proportion of the overall surgical workload. This study investigated one method to reduce the cost for a number of orthopedic surgical procedures by reducing expenses associated with SIT sterilization. Several studies have investigated the optimization and rationalization of SITs of various surgical specialties to increase operative theatre efficacy and reduce expenses [15-18]. SIT rationalization reduces the cost and reduces the physical efforts and procedure preparation time as well as tray decontamination and processing time [16]. Dyas et al. reported a reduced average trays' weight from 27 to 10 pounds (37% of the original weight) and observed a reduction in the processing time from eight to three minutes (37.5% of the original time) per tray [16]. While this study examined ENT surgical trays specifically, it shows how one group has improved efficiency and saved costs by reducing the number of instruments on the trays. Over three months, Wood et al. found potential time savings of 383.5 hours per year in tray sterilization and cost savings of $69,441 per year for general and plastic surgery in one hospital [17]. Over three months, Knowles et al. also looked at which instruments were used in surgical trays for vascular surgery and then optimized these [18]. They estimated an annual saving in sterilization of $97,444 after they removed 45.8% and 65.2% of instruments from their vascular and aortic trays, respectively. These studies from allied surgical specialties show that the rationalization of equipment can result in financial savings and improved efficiency for sterilization. While these studies were not explicitly related to orthopedic procedures and, thus, our equipment needs, it is not unreasonable to predict comparable savings and improvements within our field.

The present study used a process of examining the usability of each instrument and excluding the rarely used instruments. This reduced the number of SITs of the included procedures to almost the third of the primary number, which is reflected by a perceived yearly average saving of £61,950 (63.55% of the yearly sterilization cost). In a similar context, Cichos et al. used a lean methodology to rationalize their orthopedic SITs in one hospital over a year. They reported an annual saving of $270,976 (a 20% cost reduction) from SIT rationalization [15]. This study looked at 11 different orthopedic surgical trays over a year; 55% of instruments were removed and they had a 22% reduction in tray weight. While the total saving was higher in Cichos et al.'s study, we have saved more when we look at it as a proportion. In that study, the rationalization was seen over one year, while we have looked over three years showing that our results have been sustainable over a longer term. That study is relatable to the current study as it rationalized orthopedic surgical trays. Its findings matched other studies that came to similar conclusions - that time and money can be saved with tray rationalization [16-18].

The main challenge in the process of SIT rationalization is how to judge the usability of the surgical instrument. Instrument useability could vary according to the operating surgeon's preference. In our study, each surgical instrument for the examined trays was judged by a senior trauma and orthopedic consultant and senior scrub nurse individually, yet this may be subject to personal preference bias, which could be assessed further in the questionnaire to understand this and characterize it per operation and tray. Furthermore, to ensure the practicality and the efficiency of SIT rationalization, a three years' follow-up feedback survey was sent to the orthopedic surgeons and scrub nurses in our institution. The survey responses showed that most participants either agreed or strongly agreed that SIT rationalization is practical and reduced the unnecessary instruments.

In the questionnaire about current arthroscopic SITs, one response (6.2%) indicated that other instruments are always required, and one response (6.2%) indicated that other instruments are frequently required. However, 13 participants (75%) reported either they rarely or never need other instruments with the current arthroscopic trays, possibly relating to individual surgeon preference on equipment. Individually packed and sterilized surgical instruments can be a practical solution for occasionally needed instruments to minimize the tray number and the sterilization cost. The overall feedback received from our SIT rationalization survey was in favor of the rationalization process.

Considering the present study, we have shown that money can be saved by rationalizing the SITs within our department - £185,850 from 2,505 operations over three years. The strengths of this study are that this is a relatively simple intervention that can and has saved the department money. Unfortunately, our survey had a limited response; only 18 individuals responded to our survey with a response rate of about 76%. Increasing the response rate here from both surgical and scrub staff could help us understand the effectiveness of the tray rationalization process undertaken and help us to plan further improvements that could be made. As identified here in this project, the following steps are to think about how we can expand this to other trays within the T&O department and possibly work across the surgical specialties to think about where rationalizations can be made to make further savings.

## Conclusions

There is a real need for cost-effective health care practice nowadays more than ever before. The systemic assessment of the orthopedic surgical instruments through the rationalization process can minimize the required SITs for standard procedures. The process of orthopedic SIT rationalization is a practical and efficient approach to achieve significant savings without affecting patient safety through a reduction in sterilization costs alone.
